# Altered Static and Dynamic Spontaneous Neural Activity in Drug-Naïve and Drug-Receiving Benign Childhood Epilepsy With Centrotemporal Spikes

**DOI:** 10.3389/fnhum.2020.00361

**Published:** 2020-08-28

**Authors:** Sisi Jiang, Cheng Luo, Yang Huang, Zhiliang Li, Yan Chen, Xiangkui Li, Haonan Pei, Pingfu Wang, Xiaoming Wang, Dezhong Yao

**Affiliations:** ^1^The Clinical Hospital of Chengdu Brain Science Institute, Key Laboratory for NeuroInformation of Ministry of Education, Center for Information in Medicine, High-Field Magnetic Resonance Brain Imaging Key Laboratory of Sichuan Province, School of Life Sciences and Technology, University of Electronic Science and Technology of China, Chengdu, China; ^2^Research Unit of NeuroInformation, Chinese Academy of Medical Sciences, Chengdu, China; ^3^Department of Neurology, Affiliated Hospital of North Sichuan Medical College, Nanchong, China

**Keywords:** epilepsy, drug treatment, spontaneous neural activity, static, dynamic

## Abstract

The present study aims to investigate intrinsic abnormalities of brain and the effect of antiepileptic treatment on brain activity in Benign childhood epilepsy with centrotemporal spikes (BECTS). Twenty-six drug-naïve patients (DNP) and 22 drug-receiving patients (DRP) with BECTS were collected in this study. Static amplitude of low frequency fluctuation (sALFF) and dynamic ALFF (dALFF) were applied to resting-state fMRI data. Functional connectivity (FC) analysis was further performed for affected regions identified by static and dynamic analysis. One-way analysis of variance and *post hoc* statistical analyses were performed for between-group differences. Abnormal sALFF and dALFF values were correlated with clinical features of patients. Compared with healthy controls (HC), DNP group demonstrated alterations of sALFF and/or dALFF in medial prefrontal cortex (MPFC), supplementary motor areas (SMA), cerebellum, hippocampus, pallidum and cingulate cortex, in which the values were close to normal in DRP. Notably, sALFF and dALFF showed specific sensitivity in detecting abnormalities in basal ganglia and cerebellum. Additionally, DRP showed additional changes in precuneus, inferior temporal gyrus, superior frontal gyrus and occipital visual cortex. Compared with HC, the DNP showed increased FC in default network and motion-related networks, and the DRP showed decreased FC in default network. The MPFC, hippocampus, SMA, basal ganglia and cerebellum are indicated to be intrinsically affected regions and effective therapeutic targets. And the FC profiles of default and motion-related networks might be potential core indicators for clinical treatment. This study revealed potential neuromodulatory targets and helped understand pathomechanism of BECTS. Static and dynamic analyses should be combined to investigate neuropsychiatric disorders.

## Introduction

Benign childhood epilepsy with centrotemporal spikes (BECTS), also called Rolandic epilepsy, is the most common type of focal epilepsy in children (Berg et al., 1999), with onset ages between 1 and 14 years old (Natalio, 2010). Although most patients with BECTS go into remission from seizures after adolescence with or without drug treatment, growing evidence suggests that patients with BECTS are affected by a variety of cognitive problems, including language deficits (Overvliet et al., 2011; Besseling et al., 2013), attention dysfunctions (Kavros et al., 2008; Xiao et al., 2015) and memory impairment (Northcott et al., 2007). In addition, previous studies have suggested that antiepileptic drugs (AEDs) might have side effects on the brain activity of patients with epilepsy (van Veenendaal et al., 2015). Recently, accumulating neuroimaging evidence has indicated that distributed brain regions are involved in BECTS, including the default mode network (DMN), sensorimotor cortex and mesial temporal lobe cortex (Xiao et al., 2016; Li et al., 2017).

As a data-driven method, the amplitude of low-frequency fluctuation (ALFF) measures the magnitude of spontaneous blood oxygenation level-dependent (BOLD) activity, which depicts the energy intensity of brain activity over a period of time (Zang et al., 2007). Traditional static ALFF (sALFF) assumes that brain activity is stationary during resting-state scan and has been applied to investigate resting-state fMRI in various epilepsies. In idiopathic generalized epilepsy, the amplitude analysis of different frequencies helped to characterize brain abnormalities and provided insights into the pathomechanism of the disease (Wang et al., 2014). It was shown that increased ALFF might be related to interictal epileptic activity in mesial temporal lobe epilepsy (Zhang et al., 2010). Moreover, sALFF has been used to localize the epileptic focus of patients with BECTS (Wu et al., 2015; Chen et al., 2018). Recently, the temporal variability in resting-state fMRI has been used to capture time-varying intrinsic brain activity during scanning (Allen et al., 2014; Yan et al., 2017), which has been used to study schizophrenia (Fu et al., 2018) and major depressive disorder (Kaiser et al., 2015; Li et al., 2018). The sALFF depicts the stable activity intensity of brain regions, reflecting baseline energy consumption in maintaining fundamental brain functions. The dynamic ALFF (dALFF) depicts the plasticity and flexibility of spontaneous brain activity through the variability of energy expenditure. It has been suggested that the dynamics of brain has neurophysiological origin (Tagliazucchi et al., 2012). Thus, combination of sALFF and dALFF analysis may provide new insights into brain abnormalities and the pathophysiological mechanisms of epilepsy. Moreover, epilepsy is viewed to be a network disorder and the functional connectivity (FC) of epileptic brain network has been recognized as a crucial profile for understanding the underlying pathomechanism (Li et al., 2017). Besides, the feature of FC is of great value to the prediction of the therapeutic effect of drugs, physical intervention and other clinical treatments as well as the revelation of the corresponding internal brain network mechanism (Douw et al., 2010; Kimiskidis et al., 2014; Haneef et al., 2015). Disturbed functional interaction in several brain networks has been observed in BECTS and has to do with multiple domains of behavior of patients (Vannest et al., 2019).

Several questions need to be taken into consideration for the clinical treatment of patients with BECTS. First, what are the intrinsic abnormalities of patients with BECTS? Second, what happens to the brain activity of patients with BECTS receiving AEDs? Investigating which brain regions positively respond to the drugs would provide valuable information to help clinical neuromodulation and drug development. In the present study, drug-naïve patients (DNP) and drug-receiving patients (DRP) with BECTS were collected and compared with matched healthy controls (HC). Notably, the DNP were newly diagnosed patients who had not received antiepileptic drugs. Data driven methods (sALFF, dALFF and FC) were applied to investigate abnormal brain activity in patients in drug-naïve and drug-receiving conditions, attempting to provide some evidence to answer the above questions.

## Materials and Methods

### Participants

Fifty-two patients (mean age: 9.52 ± 1.83 years; mean months of duration: 13.09 ± 12.42; mean age of seizure onset: 8.47 ± 1.94; all right-handed, 24 females) with benign epilepsy with centrotemporal spikes were recruited at the Affiliated Hospital of North Sichuan Medical College. Twenty-eight patients were drug-naïve, and 24 patients were receiving antiepileptic drugs with good seizure control. All of the patients underwent a comprehensive clinical evaluation for the diagnosis of BECTS according to the epilepsy classification of the International League Against Epilepsy (Engel and International League Against Epilepsy, 2001). All patients in the DRP group took the drug within 1 month after diagnosis of epilepsy. In the DRP group, all patients were receiving monotherapy, with 12 on oxcarbazepine, 8 on lamotrigine, and 4 on topiramate. No patients had brain lesions, developmental disabilities, or other accompanying neurological disorders. Twenty-four healthy controls (mean age: 10.33 ± 3.70 years, all right handed, 7 girls) were recruited as a sex- and age-matched control group, and all of the controls were free from neurological and psychiatric disorders. This study was approved by the ethical committee of the Affiliated Hospital of North Sichuan Medical College according to the standards of the Declaration of Helsinki, and written informed consent was obtained from each subject and their parents.

### Data Acquisition

All subjects underwent MRI scanning in a 3T GE scanner with an eight-channel-phased array head coil (EXCITE, GE, Milwaukee, WI, United States) in the Affiliated Hospital of North Sichuan Medical College. Resting-state functional data were collected using an echo-planar imaging sequence with the following parameters: repetition time (TR) = 2000 ms, echo time (TE) = 30 ms, flip angle (FA) = 90°, slice thickness = 4 mm (no gap), data matrix = 64 × 64, field of view = 24 cm × 24 cm, voxel resolution = 3.75 mm × 3.75 mm × 4 mm, and 32 axial slices in each volume. Two hundred volumes were acquired in each scan, lasting 6 min and 40 s. Axial anatomical T1-weighted images were acquired using a 3-dimensional fast spoiled gradient echo sequence. The parameters were as follows: thickness = 1 mm (no gap), TR = 8.2 ms, TE = 3.2 ms, field of view = 25.6 cm × 25.6 cm, flip angle = 12°, data matrix = 256 × 256. There were 136 axial slices for each subject. All subjects were instructed to close their eyes and relax without falling asleep during the scan. A simple oral questionnaire was performed for each subject to ensure an awake state during the scan.

### Data Preprocessing

Resting-state fMRI data were preprocessed using the NIT software package^1^ (Dong et al., 2018). The first five volumes were discarded to ensure magnetic field stabilization. The remaining 195 volumes were slice-timing corrected and spatially realigned. Then, the functional data were spatially normalized to the standard Montreal Neurological Institute space and were resampled to 3 mm × 3 mm × 3 mm and smoothed with a Gaussian kernel (8 mm full width at half maximum, FWHM). Through aligning each time point image with the first image, six head motion parameters (three rotation parameters and three translation parameters) can be estimated. Finally, the linear trend signals, six head motion parameters, the whole brain mean signal, and white matter and cerebrospinal fluid signals were regressed out from the smoothed resting-state fMRI data using a general linear model. Any subject whose head motion exceeded 2 mm or/or 2° was excluded. Moreover, based on frame-wise displacement (FD) defined by Power (Power et al., 2012), the mean frame-wise displacement (mFD) of each subject was computed using the following formula:

mFD = $\left(\frac{1}{M-1}\right)\sum_{\text{i}=2}^{\text{M}}\left(\Delta d_{x_{i}^{1}}|+|\Delta d_{y_{i}^{1}}|+|\Delta d_{z_{i}^{1}}|+|\Delta d_{x_{i}^{2}}|+|\Delta d_{y_{i}^{2}}|+|\Delta d_{z_{i}^{2}}|\right)$, where M is the length of the time course (*M* = 195); $x_{i}^{1}$/$x_{i}^{2}$, $y_{i}^{1}$/$y_{i}^{2}$ and $z_{i}^{1}$/$z_{i}^{2}$ are translations/rotations at the *i*th time point in the *x*, *y*, and z directions, respectively; and $\Delta \,d_{x_{i}^{1}}=x_{i}^{1}-x_{i-1}^{1}$, and a similar pattern held for the others. Notably, by calculating displacement on the surface of a sphere with a radius of 50 mm, the rotations were converted from degrees to millimeters.

### Static and Dynamic Amplitude of Low Frequency Fluctuations

For each voxel in the whole brain, a fast Fourier transform was first performed to convert the time series to the frequency domain. Then, the averaged square root of the power spectrum across an frequency band was calculated as the ALFF value (Zang et al., 2012). In the present study, we selected the frequency band 0.01–0.08 Hz from the full frequency range (0.01–0.25 Hz) to calculate the sALFF for each group, and the sALFF value of each voxel was divided by the global mean of sALFF values.

**FIGURE 1:**
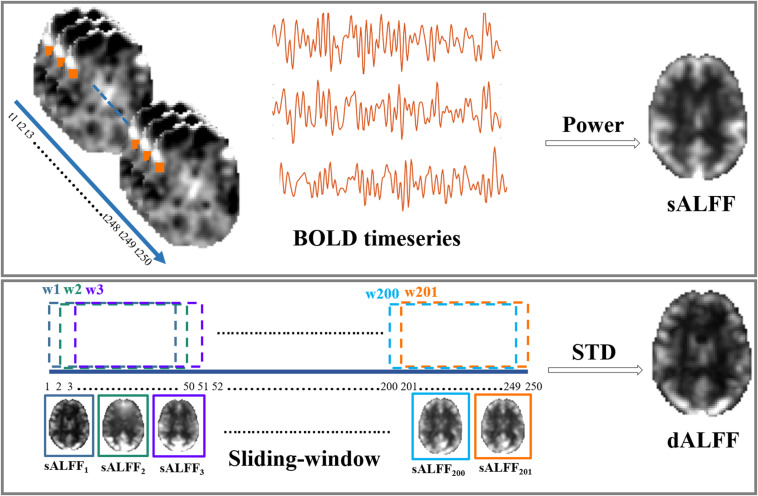
Illustration of static and dynamic ALFF calculation.

For the dynamic feature, a sliding-window approach was adopted in the present study to examine the temporal variability in ALFF over the duration of the scan. In the present study, a 100 s window length (*L* = *50 TR*) and 2 s steps (*S* = *1 TR*) were used, considering that the window length should be in line with the commonly identified slowest frequency of the BOLD signal (Leonardi and Van De Ville, 2015; Zalesky and Breakspear, 2015). Thus, we high-pass filtered the time series at 0.01 Hz. Briefly, for a given voxel, the time series, consisting of 195 timepoints (*F* = 195 *TR*), was segmented at each time point by obtaining *146 (W* = *F-L* + *1)* sequential time windows. The sALFF was calculated within each segmented window, thus generating 146 sALFF values for every voxel. Then, the standard deviation (SD) across 146 continuous sALFF values was calculated to represent the temporal variety of brain activity. The same calculation steps were performed for every voxel in the whole brain to acquire the dALFF maps. Finally, the SD maps were z-standardized across all the voxels for the following statistical analysis. A rough illustration of sALFF and dALFF was shown in Figure 1.

### Functional Connectivity Profiles of Affected Regions

Based on the affected regions revealed by above sALFF and dALFF analyses, further FC was conducted. Before FC calculation, the global signal was regressed out from the previously preprocessed data. First, each region identified above was viewed as a seed. We calculated the mean time series of a sphere with a radius of 6 mm centered on the peak voxel and used it as a surrogate time series to correlate with whole-brain voxels. Besides, cross-correlation between these regions was calculated using Pearson correlation and followed by Fish-Z transformation. Second, we identified the affected regions revealed by both dynamic and static analysis as core affected regions. The FC architectures of these core affected regions were investigated in a whole-brain manner. That is, FC was computed between each core affected region and every other voxel of the whole brain.

### Statistical Analysis

First, one-sample *t*-tests were performed on sALFF and dALFF maps to evaluate the within-group patterns across the three groups (DRP, DNP, and HC). Then, one-way analysis of variance (ANOVA) was used to detect the differences among the three groups, with age, gender, and mFD values of head motion as nuisance variables. Tukey-kramer *post hoc* analysis was performed to investigate pairwise between-group differences. Furthermore, we also performed partial correlation analysis to detect the relationship between the value of sALFF and dALFF in brain regions with significant between-group differences and the clinical features, including onset age, and illness duration, controlling for the effects of gender. ANOVA and *post hoc* statistical analyses were also conducted for the FC profiles.

### Validation

Here, because head motion could have a significant effect on dynamic features, to validate the findings, the sALFF and dALFF analyses were replicated in a subgroup with a stricter requirement of head motion (mFD < 0.2), consisting of 18 DRP, 19 DNP and 15 HC. Detailed demographic data are illustrated in Supplementary Table 1. The same statistical analyses were performed to detect between-group differences, as mentioned above. To illustrate the consistency in the findings between the whole dataset and the subgroup dataset, Pearson’s correlation coefficients were calculated between the statistical t-maps of the two datasets.

## Results

Four of the 52 recruited patients with BECTS were excluded from the sALFF and dALFF analyses because of excessive head motion, including two DRP and two DNP. There were no significant differences among the three groups for age. However, the differences of gender were observed between DRP and DNP (*p* = 0.04), between DNP and HC (*p* = 0.02). Besides, the mFD values between DNP and HC showed difference (*p* = 0.03). In addition, the onset age between DNP and DRP did not show a significant between-groups difference (two sample *t*-test, *p* = 0.22). The DRP demonstrated a significantly longer illness duration and lower seizure frequency than DNP (*p* < 0.001). Detailed clinical and demographic information are shown in Table 1.

**TABLE 1:** Demographic, clinical and neuropsychological features of the subjects.

**Characteristics**	**DRP (*n* = 22) Mean ± SD**	**DNP (*n* = 26) Mean ± SD**	**HC (*n* = 24) Mean ± SD**	***P*_1_ value (DRP-DNP)**	***P*_2_ value (DRP-HC)**	***P*_3_ value (DNP-HC)**
Age (year)	9.68 ± 1.73	9.38 ± 1.97	10.33 ± 3.76	0.59^*a*^	0.47^*a*^	0.27^*a*^
Gender (female/male)	7/15	16/10	7/17	**0.04^*b*^**	0.85^*b*^	**0.02^*b*^**
Illness duration (month)	20.41 ± 12.32	6.91 ± 9.05	–	**<0.0001^*a*^**	–	–
Onset age (year)	8.09 ± 2.06	8.79 ± 1.86	–	0.22^*a*^	–	–
Mean FD (mm)	0.14 ± 0.06	0.15 ± 0.1	0.20 ± 0.12	0.50^*a*^	**0.03^*a*^**	0.13^*a*^
Seizure frequency(time/month)	0.32 ± 0.91	2.62 ± 1.88	–	**<0.0001^*a*^**	–	–

*^*a*^The *p* value was obtained by a two-sample two-tail *t* test. ^*b*^The *p* value was obtained by a χ^2^ test. The bold values indicate significant between-group differences.*

**FIGURE 2:**
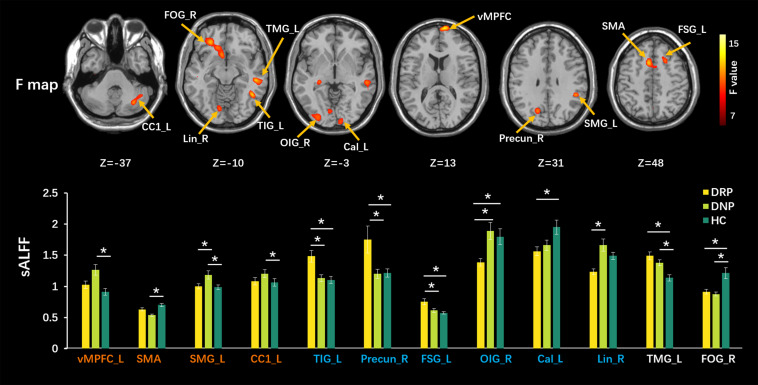
Comparisons of sALFF among three groups. The upper row shows F statistical maps and the lower row shows significant Tukey-kramer *post hoc* results (*p* < 0.01). The orange names indicate regions responding to AEDs positively, which shows significant difference from HC in DNP and closed value to HC in DRP. The blue names represent regions with additional changes in DRP, that is, no different between DNP and HC. The white names represent regions that did not respond to AEDs (the DNP and DRP have abnormalities in these regions). The asterisks (*) show a significant difference between groups. R: right, L: left.

High dALFF values had a similar distribution to that of sALFF values in the three groups. Detailed information (one-sample *t*-test) for each group is shown in Supplementary Figure 1. Significantly altered sALFF values were detected among the three groups using ANOVA and *post hoc* analyses, with a significance threshold of *p* < 0.005 with voxel number >100. Compared with HC, some alterations were only observed in the DNP group (Figure 2), including increased sALFF in the ventral medial prefrontal cortex (vMPFC), left supramarginal gyrus (SMG_L) and the left cerebellar crus1 (CC1_L) and decreased sALFF in the supplementary motor area (SMA). The sALFF in the left supramarginal gyrus (SMG_L) in DRP was similar to that in the HC group and lower than that in the DNP group. In addition, the DRP group demonstrated increased sALFF in the left inferior temporal gyrus (TIG_L), right precuneus (Precun_R), and left superior frontal gyrus (FSG_L) relative to the DNP and HC groups (Figure 2). DRP showed the lowest sALFF in the right inferior occipital gyrus (OIG_R), right lingual gyrus (Lin_R) and calcarine sulcus (Cal) among the three groups (Figure 2), and the DNP and HC did not demonstrate significant differences. In addition, both groups of DNP and DRP demonstrated increased sALFF in the left middle temporal gyrus (TMG_L), with higher values being observed in the DRP group. The sALFF in the right orbital frontal gyrus (FOG_R) was decreased in DNP and DRP relative to HC and was indiscriminate between these two groups (Figure 2).

**FIGURE 3:**
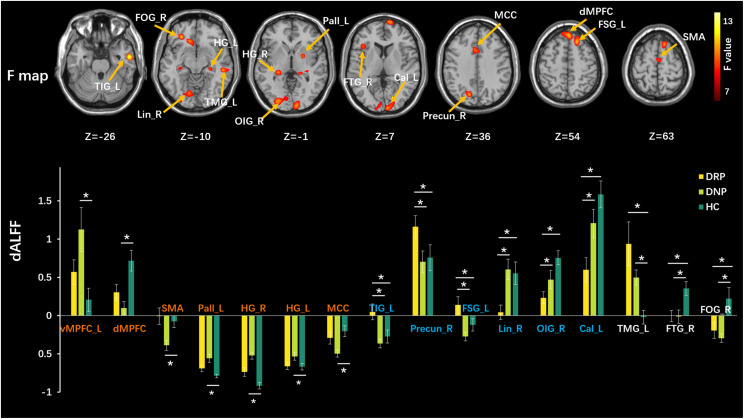
Comparisons of dALFF among three groups. The upper row shows F statistical maps and the lower row shows significant Tukey-kramer *post hoc* results (*p* < 0.01). The orange names indicate regions that positively respond to AEDs, the blue names represent regions with additional changes in patients with treatment and the white names represent regions that did not respond to AEDs. The asterisks (*) show significant difference betweens groups. R: right, L: left.

**FIGURE 4:**
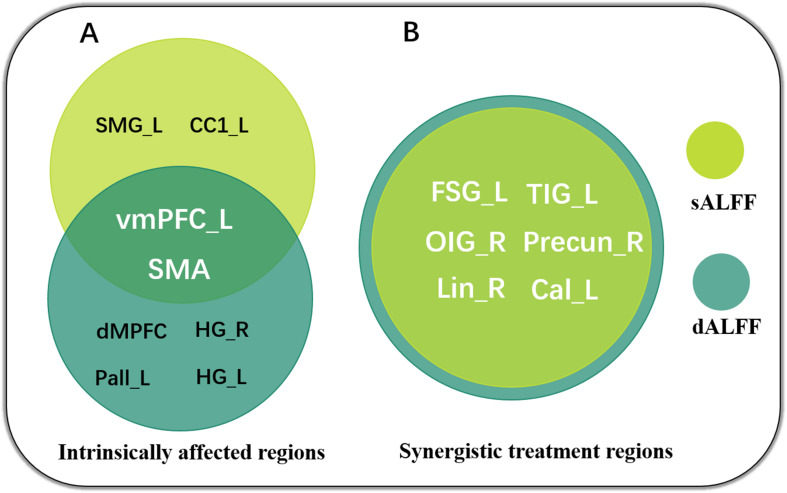
Summary of sALFF and dALFF findings, revealing intrinsically affected regions **(A)** and synergistic treatment regions **(B)** in the patients with BECTS. Notably, the sALFF and dALFF concordantly highlight the crucial role of vmPFC and SMA in AED therapy.

We used a sliding-window method to characterize the dynamic ALFF in the frequency band 0.01–0.08. Significant differences in dALFF were observed among the three groups (one-way ANOVA, *p* < 0.005, and voxel number >100) (Figure 3). Compared with HC, the DNP group showed increased dALFF in left vMPFC and SMA and decreased dALFF in the dorsal mesial prefrontal cortex (dMPFC), left pallidum (Pall_L), right and left hippocampus gyrus (HG_R and HG_L), while DRP did not demonstrate any alterations (Figure 3). Notably, DRP were not significantly different from HC in these regions. In addition, both DNP and DRP showed increased dALFF in TIG_L, Precun_R and FSG_L and decreased dALFF in Lin_R, OIG_R and Cal_L (Figure 3). Notably, no differences between DNP and HC were observed in the six regions. Compared with HC, both the DNP and DRP showed increased dALFF in TMG_L and decreased dALFF in right frontal triangle gyrus (FTG_R) and FOG_R, which were not different between DRP and DNP (Figure 3). A straightforward summary of sALFF and dALFF findings is shown in Figure 4.

**FIGURE 5:**
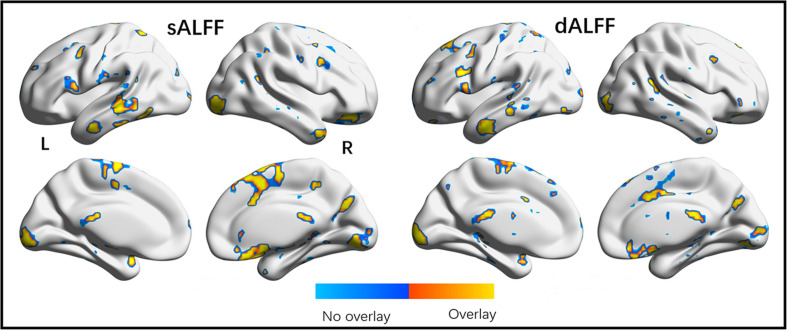
Overlap of significant regions observed in origin and validation tests. The validation dataset was a subgroup in origin dataset with extremely small head motion (mFD < 0.2). High overlap of brain regions shown in two statistical analyses validates the findings demonstrated in the present study.

Moreover, the subgroups with mFD < 0.2 demonstrated almost the same results as those of the whole dataset. The correlation coefficient of the F maps of the two datasets reached 0.77. The correlation of *post hoc* statistical maps between two datasets reached 0.84, 0.83, and 0.90 for DRP-DNP, DNP-HC and DNP-HC comparisons, respectively. The significant F maps of the two datasets were overlapped to illustrate high spatial similarity between the two datasets (Figure 5).

**FIGURE 6:**
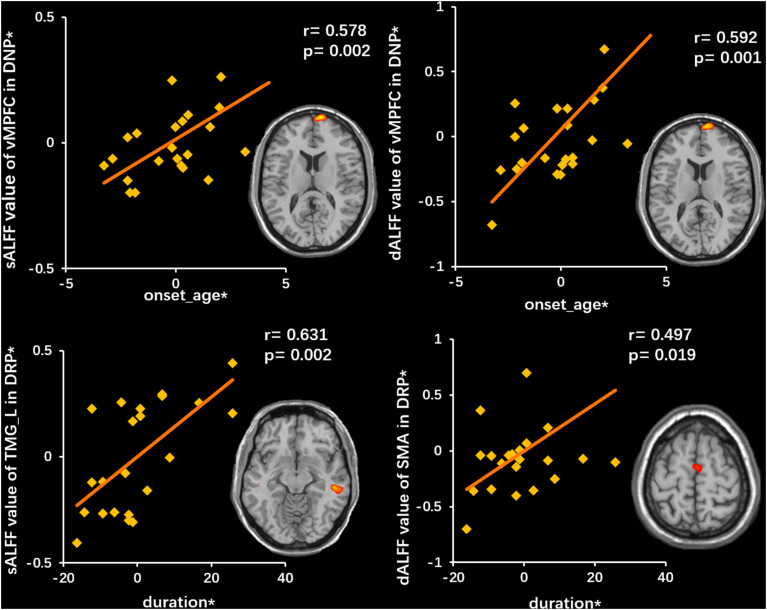
Correlations between neuroimaging variates and clinical features. The top row illustrates a positive correlation between sALFF (left) and dALFF (right) of vMPFC and onset age in DNP. The bottom row illustrates a positive correlation between sALFF (left) of TMG_L and dALFF (right) of SMA and duration. The asterisk (*) indicates coordinate values controlling for the influence of gender.

The duration of the illness in the DRP group showed a positive correlation with the sALFF in TMG_L (*r* = 0.631, *p* = 0.002) and with the dALFF in the SMA (*r* = 0.497, *p* = 0.019). In addition, the onset age of DNP was positively related to the sALFF (*r* = 0.578, *p* = 0.002) and the dALFF (*r* = 0.592, *p* = 0.001) in the vMPFC (Figure 6).

**FIGURE 7:**
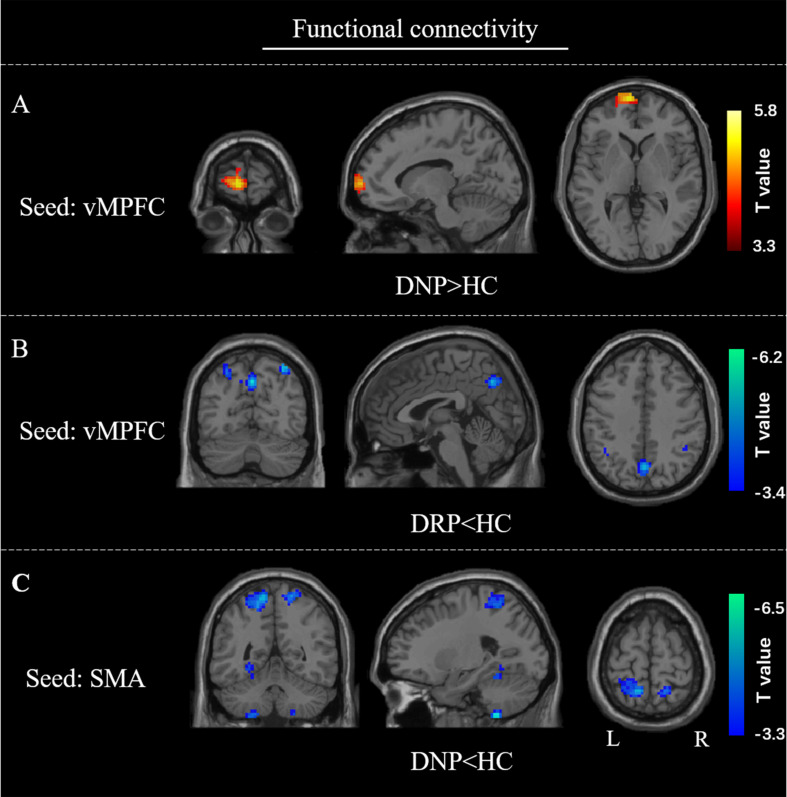
FC alterations of vMPFC and SMA. Compared with HC, **(A)** increased FC of vMPFC in DNP; **(B)** decreased FC of vMPFC in DRP; **(C)** decreased FC of SMA in DNP.

For the cross correlation between of pairs of affected regions, no significant (*p* < 0.001) alteration was found in current study. In the voxel-wise FC analysis, increased FC within vMPFC was found in DNP relative to HC (*p* < 0.001). Besides, decreased FC between vMPFC and posterior DMN regions (precuneus and inferior parietal gyrus) was observed in DRP compared with HC (Figures 7A,B). Moreover, the DNP showed decreased FC between SMA and precuneus, superior parietal gyrus and cerebellum (Figure 7C). Detailed information of brain regions with significant between-group differences was shown in Table 2.

**TABLE 2:** Brain regions showing significant alteration of FC in BECTS.

**Regions**	**MNI coordinates**	**Peak T value**	**Number of voxels**
**vMPFC (DNP > HC)**			
Frontal_Sup_Medial_L	−6 66 3	4.80	77
Frontal_Sup_L	−15 69 5	4.17	
**vMPFC** (DRP < HC)			
Parietal_Inf_L	−39 −48 33	−3.69	38
Precuneus_L	0 −69 42	−4.44	92
Precuneus_R	2 −66 42	−4.27	
Parietal_Sup_L	−27 −69 54	−3.85	34
Parietal_Sup_R	33 −75 54	−4.66	33
**SMA (DNP < HC)**			
Cerebelum_8_R	21 −45 −57	−4.52	23
Parietal_Sup_L	−12 −51 66	−4.36	174
Precuneus_L	−14 −51 63	−4.07	
Parietal_Sup_R	15 −54 69	−4.04	81

## Discussion

This study investigated spontaneous neural activity from static and dynamic aspects in BECTS patients with and without drug treatment. First, naïve patients showed abnormal activity in MPFC, hippocampus, SMA, pallidum and cerebellum, and drug-treated patients demonstrated a normalization effect on these regions. These findings suggested potential intrinsically affected regions in BECTS and indicated that AEDs could have a positive therapeutic effect by affecting the activity of the abovementioned regions. In addition, disrupted activity in precuneus, occipital visual cortex and lateral temporal regions was only observed in DRP, which might imply specific synergistic normalization effects related to AEDs. Moreover, specific disruption in DNP suggested that the abnormal FC in default and motion-related networks might be disease-intrinsic impairment. Distinct FC patterns in DRP further implied a potential therapeutic effect on these networks. In summary, the present study investigated intrinsic dysfunctions related to BECTS and the effects of AEDs on the brain activity of patients, providing insights into the pathomechanism of the disease. Finally, this study also indicated the necessary combination of static and dynamic activity in studying epilepsy.

The DNP showed altered sALFF and dALFF in the vMPFC, hippocampus and SMA, and the DRP demonstrated values similar to those of HC in this study. The vMPFC and hippocampus are the core nodes of the well-known DMN, which has been widely reported to be associated with various epilepsies (Li et al., 2017). The DMN was identified as contributing to the generation and propagation of epileptic activities (Gotman et al., 2005; Clemens et al., 2016) and cognitive impairment (Gusnard et al., 2001). Specifically, it has been demonstrated that the MPFC is an important node in epileptic network. High activity power and high variability of energy consumption of MPFC in newly diagnosed patients relative to HC might provide an energy-excess and over-flexible circumstance facilitating seizure. The higher sALFF and dALFF in the vMPFC observed with older onset age in the present study further indicated the crucial role of vMPFC activity in BECTS. Besides, both the DNP and DRP showed significant FC with vMPFC further emphasized its crucial role in the disease. The FC between vMPFC and distinct DMN regions observed in both patient groups implied that the DMN might be potential area where the effects of drug therapy occur. In addition, the hippocampus was recognized as an important brain region for memory function. Previous studies have suggested memory deficits in patients with BECTS (Kim et al., 2014). Present study revealed no static power change but increased variability of hippocampus activity, which implied that the hippocampus is more prone to abnormal alterations in dynamic characteristics. The change toward normality in the hippocampus in DRP in the present study might imply an improvement in cognitive function in patients with treatment. Since this study did not evaluate the memory function, this explanation is speculation based on previous studies. Moreover, it was suggested that the SMA was responsible for motor abnormalities in patients with epilepsy (O’Muircheartaigh et al., 2012). It was easy to link the changes in the SMA to the improvement in clinical motor symptoms in patients with seizure control after treatment. We found decreased FC between SMA and parietal and cerebellar regions in naïve patients supported the motor dysfunction elicited by epilepsy and suggested a positive response of SMA to drug treatment. The significantly positive correlation between dALFF in SMA and the illness duration also supported the effective therapy of AEDs on SMA. Our findings provided evidence indicating that the MPFC, hippocampus and SMA were affected by the disease itself. The ALFF values close to those of HC in these regions in the DRP further indicated that AEDs might work by changing the activity of these brain regions, which implied potential targets for neuromodulation and/or drug treatment.

In the present study, increased sALFF in the left cerebellum and decreased dALFF in the left pallidum were also observed in the DNP, and no difference was observed in the two regions between the DRP and HC. This finding showed that sALFF and dALFF had different sensitivities in detecting abnormal activities in different brain regions. Increased sALFF in cerebellum in DNP implied that excessive cerebellar activity might be related to seizures. Besides, decreased nodal efficiency and regional homogeneity and increased gray matter volume of cerebellum have been reported in patients with BECTS (Luo et al., 2015; Zeng et al., 2015; Ji et al., 2017), indicating the involvement of the cerebellum in BECTS. Evidence from animal studies has indicated that the cerebellum contributes to the termination of epileptic activities (Kros et al., 2015). Neuroimaging studies in humans also supported a potential modulatory effect of the cerebellum on the involved brain network. In addition, a synchronous EEG-fMRI study suggested a modulating effect of the basal ganglia on epileptic activities (Luo et al., 2012). Altered basal ganglia–cortical FC has been revealed in focal epilepsy (Dong et al., 2016). The decreased dALFF of basal ganglia observed here might be the adaptive alteration of flexibility to an uncontrolled epileptic environment. Both the cerebellum and basal ganglia were viewed as important modulators of epileptic activities. The basal ganglia and cerebellum are interconnected and have been demonstrated to be closely related to the thalamocortical circuit in patients with generalized epilepsy (Bostan et al., 2010; Hu et al., 2017). Although BECTS is a type of focal epilepsy, the distributed brain network has been reported to be affected, which might be modulated by the basal ganglia and cerebellum. Meanwhile, the DRP demonstrated similar sALFF values in the cerebellum and dALFF values in basal ganglia as those of HC, which indicated potential therapeutic effects and further supported the potential modulatory roles of the two regions. sALFF and dALFF provided complementary information to reveal the involvement of the cerebellum and basal ganglia in BECTS, contributing to fully understanding the pathological mechanism of BECTS.

In the present study, additional abnormalities in the occipital visual cortex, lateral temporal regions and the right precuneus were shown in the DRP, which might be interpreted in two possible ways. On the one hand, it was recognized that the long-term use of AEDs might cause some chronic brain damage (van Veenendaal et al., 2015), and more serious consequences might arise from receiving AEDs during growth and development as a child (Loring and Meador, 2004). On the other hand, because the mechanisms by which drugs work are complex, there is no evidence to deny that these adaptive changes might have synergistic normalization effects under the influence of drugs. Based on accumulated previous studies, these regions are functional or anatomic linked with the regions identified to have positive response to drug therapy (Liao et al., 2011; Luo et al., 2011; Liu et al., 2017). The brain is a large-scale network which dynamically regulates the information interaction between various systems to maintain a balance state (Hutchison et al., 2013). In our opinion, we tended to infer that these regions might play some synergistic roles in therapeutic efficacy, such as compensatory changes. In addition, both sALFF and dALFF revealed that drug treatment did not work on the left middle temporal gyrus, the right orbital frontal gyrus and the right frontal triangle gyrus. Whether these sustained abnormalities suggest a deficiency of AEDs or implied non-core roles of these regions in the epileptic network requires further research.

In addition, there are some limitations in the present study. First, the best experimental design for drug efficacy studies should be a longitudinal study, but this study is a cross-sectional study. Recruiting two independent patient groups with and without drug treatment to investigate the therapy effect made it difficult to exclude the confounding effects related to variability between subjects. Therefore, to address these issues, a cohort study needs to be conducted in the future. Second, despite widely reported cognitive impairment in BECT in previous studies, the lack of cognition assessment weaken the clinical meaning of the present study to some extent. Third, the gender was different between groups. In the present study, the gender was regressed out as a nuisance variate in the linear regression model of statistic analysis. Still, we can’t completely rule out the potential impact of gender differences on current results.

## Conclusion

The present study adopted sALFF and dALFF to characterize abnormal brain activities in the DNP and DRP groups compared with the HC group. Low-frequency neural oscillations in the MPFC, hippocampus, SMA, basal ganglia and cerebellum were only altered in drug-naïve patients, not in the treated group; thus, these alterations were inferred to be induced by the disease itself. FC profiles further suggested the crucial role of DMN and motion-related regions for the treatment of epilepsy. This finding revealed effective therapeutic targets and provided additional information for understanding the pathomechanism underlying BECTS. Notably, sALFF and dALFF demonstrated specific sensitivity in detecting abnormal activity in the cerebellum and basal ganglia, respectively, indicating the necessity of combining the two methods in epilepsy research.

## Data Availability Statement

The datasets generated for this study are available on request to the corresponding author.

## Ethics Statement

The studies involving human participants were reviewed and approved by Affiliated Hospital of North Sichuan Medical College. Written informed consent to participate in this study was provided by the participants’ legal guardian/next of kin.

## Author Contributions

CL was responsible for study design. YH, YC, and ZL collected the data. SJ, HP, and PW performed data analysis and article writing. XL provided the methodological advice. CL and DY supervised the conduct of the study. SJ wrote the manuscript. XW proofread the manuscript. All authors contributed to the article and approved the submitted version.

## Conflict of Interest

The authors declare that the research was conducted in the absence of any commercial or financial relationships that could be construed as a potential conflict of interest.

## Footnotes

1 http://www.neuro.uestc.edu.cn/NIT.html
