# Supplementary material for: Altered Static and Dynamic Spontaneous Neural Activity in Drug-Naïve and Drug-Receiving Benign Childhood Epilepsy With Centrotemporal Spikes
Source: Front Hum Neurosci. 2020 Aug 28;14:361. doi: 10.3389/fnhum.2020.00361 (PMC7485420; doi:10.3389/fnhum.2020.00361)
Supplement: Supplementary file 1 [file Table_1.DOCX]

Table S1: Demographic and clinical characteristics of participants with FD<0.2.

| Characteristics | Medical  (n=18)  Mean ± SD | Nonmedical  (n=19)  Mean ± SD | HC  (n=15)  Mean ± SD | P value |
| --- | --- | --- | --- | --- |
| Age (year) | 9.69±1.65 | 9.66±1.98 | 10.20±4.58 | 0.849^a^ |
| Gender(female/male) | 7/11 | 13/6 | 5/10 | 0.79^b^ |
| Duration (month) | 21.61±12.75 | 7.24±10.04 | - | 0.001^c^ |
| Onset age (year) | 8.03±1.99 | 9.08±1.83 | - | 0.11^c^ |
| FD (mm) | 0.118±0.037 | 0.105±0.030 | 0.128±0.039 | 0.191^a^ |

a. The P value was obtained by one-way ANOVA test.

b. The P value was obtained by a χ2 test.

c. The p value was obtained by a two-sample two-tail t test.


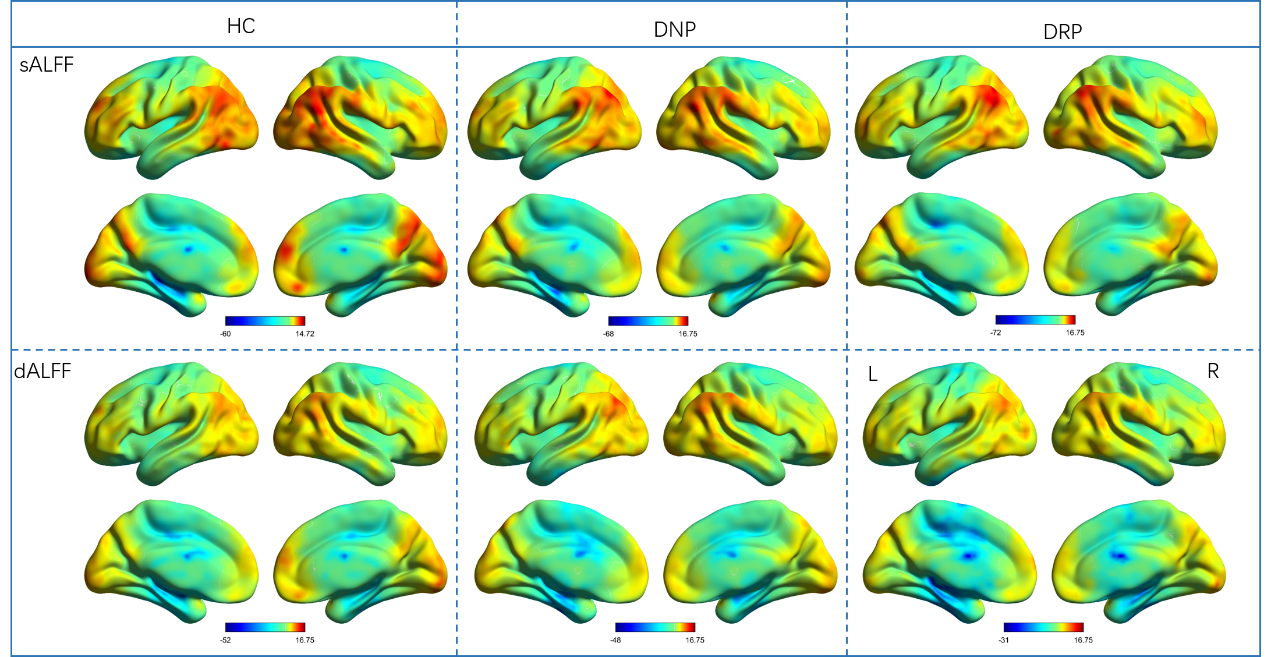


Figure S1. Spatial distribution of sALFF and dALFF within three groups.
